# Helminth diversity and seasonality of *Angiostrongylus cantonensis* in hedgehogs from Mallorca[Fn FN1]

**DOI:** 10.1051/parasite/2024069

**Published:** 2024-11-06

**Authors:** Sofia Delgado-Serra, Jessica Sola, Miquel Puig Riera, Sebastià Jaume-Ramis, Ana Sanz-Aguilar, Claudia Paredes-Esquivel

**Affiliations:** 1 Parasitology and Mediterranean Ecoepidemiology Research Group, University of the Balearic Islands 07122 Palma Spain; 2 Consorci per a la Recuperació de la Fauna de les Illes Balears (COFIB) 07002 Santa Eugènia Spain; 3 Animal Demography and Ecology Unit, IMEDEA (CSIC-UIB) Miquel Marquès 21 07190 Esporles Spain; 4 CIBER de Enfermedades Infecciosas, Instituto de Salud Carlos III C/Monforte de Lemos 3–5, Pabellón 11, Planta 0 28029 Madrid Spain

**Keywords:** *Angiostrongylus cantonensis*, Nematoda, Acanthocephala, Seasonality, Wildlife, Cestoda

## Abstract

Sentinel surveillance plays a critical role in monitoring pathogen circulation, assessing potential threats for species conservation, and evaluating the risk of spillover to human populations. This study provides a comprehensive exploration of helminth parasites in the Mediterranean-distributed hedgehog species *Atelerix algirus* in Mallorca, Balearic Islands. Using an integrated approach that combines necropsies and morphological and molecular identifications using the *COI* gene, we identified 11 helminth taxa in 135 hedgehogs, representing half of those that died at the local wildlife hospital in Mallorca between 2019 and 2022*.* We report an overall *A. cantonensis* prevalence of 11.5% and confirm the first case of a subclinical neuroangiostrongyliasis infection in a wildlife host. Infection prevalences over the year revealed that only two species, the nematode *A. cantonensis* and the cestode *Mathevotaenia* sp., had a seasonal pattern, with most *A. cantonensis* cases occurring in autumn and, to a lesser extent, *Mathevotaenia* sp. cases in winter. This pattern is probably due to the higher abundance and greater activity of snails and slugs (intermediate hosts) during these seasons, with important implications for public health and strategies for prevention of neuroangiostrongyliasis. Other key findings include a high prevalence (88.1%) of the lungworm *Crenosoma striatum* and detection of the acanthocephalan *Moniliformis saudi* for the first time in *A. algirus*. We anticipate that our study will facilitate surveillance efforts and clarify species identities in future studies. Given the lethal effects of *A. cantonensis* infection in hedgehogs, further studies are needed to evaluate the threat this parasite represents to European wildlife.

## Abstract (fr)

La surveillance sentinelle joue un rôle essentiel dans le suivi de la circulation des agents pathogènes, l’évaluation des menaces potentielles pour la conservation des espèces et l’évaluation du risque de propagation aux populations humaines. Cette étude, réalisée à Majorque dans les îles Baléares, fournit une exploration complète des helminthes parasites chez l’espèce de hérisson *Atelerix algirus*, qui est répartie dans les pays méditerranéens. En utilisant une approche intégrée qui combine des autopsies et des identifications morphologiques et moléculaires basées sur le gène *COI*, nous avons identifié 11 taxons d’helminthes chez 135 hérissons, représentant la moitié de ceux qui sont morts à l’hôpital local pour la faune sauvage de Majorque entre 2019 et 2022. Nous rapportons une prévalence globale d’*Angiostrongylus cantonensis* de 11,5 % et confirmons le premier cas d’infection par neuroangiostrongylose subclinique chez un hôte sauvage. Les prévalences d’infection au cours de l’année ont révélé que seules deux espèces, le nématode *A. cantonensis* et le cestode *Mathevotaenia* sp., présentaient un schéma saisonnier, la plupart des cas d’*A. cantonensis* survenant en automne et ceux de *Mathevotaenia* sp., dans une moindre mesure, en hiver. Ce schéma est probablement dû à la plus grande abondance et à la plus grande activité des escargots et des limaces (hôtes intermédiaires) pendant ces saisons, avec des implications importantes pour la santé publique et les stratégies de prévention de la neuroangiostrongylose. D’autres résultats clés incluent une prévalence élevée (88,1 %) du nématode pulmonaire *Crenosoma striatum* et la détection de l’acanthocéphale *Moniliformis saudi* pour la première fois chez *A. algirus*. Nous prévoyons que notre étude facilitera les efforts de surveillance et clarifiera les identités des espèces dans les études futures. Compte tenu des effets mortels de l’infection par *A. cantonensis* chez les hérissons, d’autres études sont nécessaires pour évaluer la menace que ce parasite représente pour la faune européenne.

## Introduction

One of the main challenges we will confront in the 21st century is the increase in infectious diseases that affect both humans and wildlife [62]. Zoonotic diseases account for 60% of the known emerging human infections, 72% of which originate in wildlife [44]. In recent years, there has been a significant surge in interest regarding wildlife parasites because of their role in the epidemiology of emerging infectious diseases [86]. Furthermore, there is a growing number of reports on wildlife parasites infecting domestic animals, thereby increasing the risk of spillover and spread to human populations [66]. In this regard, the use of wildlife as sentinels has emerged as a promising and effective strategy for monitoring pathogen circulation in nature; however, this type of surveillance is still underused in most regions [37].

Among parasites, helminths are more likely to be associated with zoonotic diseases than other groups, with approximately 95% of human helminth infections having zoonotic origins [85]. While most research on helminths has focused on this aspect, metazoan parasites also play a crucial role in ecosystems as they can negatively impact host fitness by affecting reproduction and ultimately survival [1, 68]. Therefore, we still need to understand the fitness effects of helminths in various wild animals [71]. Additionally, our knowledge of most wildlife parasites remains limited, and many lack molecular characterisation [33], which can hinder surveillance efforts.

Hedgehog populations are declining in Europe. This is particularly evident in *Erinaceus europaeus*, populations of which are in decline with reductions documented in the Netherlands [41], Sweden [48] and the United Kingdom [89]. Similarly, *Atelerix algirus*, the North African hedgehog, has also been reported to be in decline in mainland Spain [32]. The primary threats identified for both species include accidental road kills, habitat destruction and their capture to be kept as pets [6]. Despite the North African hedgehog being classified as a Least Concern species by the International Union for Conservation of Nature (IUCN), the available information is considered insufficient to properly assess population tendencies, and close monitoring is recommended [6]. On the island of Mallorca in the Balearic Islands (western Mediterranean), these mammals are one of the most common rescued vertebrate species in the island’s wildlife rehabilitation hospital, a situation that appears to be common throughout the continent regarding *E. europaeus* [36, 59]. *Atelerix algirus* is distributed throughout North Africa, the Spanish Mediterranean coast and the Canary Islands [6]. In the Balearic Islands, it occurs below 600 m elevation, in the garrigue near the sea and in farmlands, gardens and parks [2]. Hedgehog offspring are born between June and October, with litters ranging in number from one to three hoglets. This nocturnal mammal occasionally hibernates between January and March. In warm winters, hibernation episodes are short, lasting only a few days or even less [2].

Hedgehogs can harbour zoonotic pathogens, including helminths, bacteria, viruses, protozoa and fungi [45]. In the case of helminths, various species of trematodes, nematodes, cestodes and acanthocephalans have been detected in these mammals and some of them are noteworthy because of their zoonotic potential [72]. The hedgehog’s diet includes insects, earthworms, mealworms, slugs, snails and small vertebrate eggs [39]. Invertebrates can serve as intermediate hosts for many parasites with indirect life cycles [33]. Because of their wide distribution, relative abundance, generalist diet and their sensitivity to habitat changes, pathogens and pollutants, hedgehogs can be good bioindicators of ecosystem health [40]. In this sense, they can serve as sentinel species to monitor the occurrence of helminth parasites in natural ecosystems, with implications for both wildlife and human health [22].

In Mallorca, *A. algirus* has proven to be an effective sentinel species for monitoring zoonotic parasites such as *Angiostrongylus cantonensis*, commonly known as the rat lungworm [22, 42]*.* Its suitability as a sentinel species stems from its ubiquity and high abundance on the island, coupled with its susceptibility to this brain-infecting nematode, which can cause lethal infections in this species [22, 42]. Given the unknown long-term impact of this zoonotic parasite on hedgehog conservation and the limited information available about its helminth fauna, our aim was to determine the prevalence and seasonality of *A. cantonensis* in *A. algirus*. Additionally, we sought to evaluate its role as a sentinel species for other helminths. Using an integrated approach that combined both morphological and molecular techniques, we have comprehensively characterised the helminth parasites found in North African hedgehogs from the Mediterranean island of Mallorca. Our research not only provides novel insights into the molecular identity, prevalence and seasonality of helminths parasitizing hedgehogs, but also underscores the potential risks to human health associated with the presence and circulation of this potentially zoonotic species.

## Materials and methods

### Ethics statement

The research protocols for specimen collection and manipulation in this study were approved in advance by the biosafety committee of the University of the Balearic Islands CBS-AB 07/2020 and the *Conselleria de Medi Ambient I territory* reference TRA 04/2023. This study adhered to the regulations of the Balearic government.

### Study site and parasite collection

This study was conducted in Mallorca (2°39′03″ E, 39°34′14″ N), the largest island of the Balearic archipelago, in the western Mediterranean Sea. The island’s climate is typically Mediterranean, characterised by mild and stormy winters, dry hot summers and an annual rainfall of 450 mm per year.

Between 2013 and 2022, 273 North African hedgehogs died and were brought to the local Wildlife Rehabilitation Centre the *Consorci per a la Recuperació de la Fauna de les Illes Balears*, herein referred to as the COFIB wildlife hospital. An authorisation to examine the carcasses for scientific purposes was obtained (CBS-AB 07/2020). We conducted examinations on 135 randomly selected carcasses. In cases involving traumatic injuries or decomposition, certain organs could not be subjected to necropsy because of their condition and prevalence rates were calculated based on the total number of organs examined. Specimen collection was constrained by the available resources and limitations of the COFIB staff. During the summer, the primary focus of the centre is directed towards birds and reptiles included in the Balearic catalogue of threatened species, which arrive in large numbers. Therefore, collection of hedgehog specimens was restricted to the autumn, winter and spring months.

Necropsies were conducted in a Biosafety Level 2 facility, following the guidelines of the University of the Balearic Islands. The dissection of each organ was performed under a magnifying glass (5×). In total, we examined the following organs: brains (96), nasal sinuses (89), lungs (109), oesophagi (74), stomachs (73), intestines and peritonea (87), kidneys (19), livers (20), gallbladders (22) and hearts (86). Brain parasites were collected using the tissue digestion technique of Arango-Colonna *et al.* [8]. Helminth parasites collected were preserved in 70% ethanol at −18 °C.

### Morphological identifications

Nematodes and acanthocephalans were clarified using lactophenol (25% phenol, 25% lactic acid, 25% glycerine, 25% distilled water) until the internal structures were visible. Acanthocephalans and cestodes were fixed with Bouin’s fixative for 48 hours and stained for 24 hours with Langeron’s chlorhydric carmine [15]. The stained parasites were mounted on glass slides using DPX before being observed under the microscope. Identification of the parasites relied on the keys and morphological descriptions of Dougherty [26], Vieira *et al.* [88] and Stunžėnas and Binkienė [83] for *Crenosoma* species; Butterworth and Beverley-Burton [14], Hoa [38] and Moravec *et al.* [60, 61] for Capillariidae; Amin *et al.* [3] for *Plagiorhynchus* spp.; Amin *et al.* [4] and Van Cleave [18] for *Moniliformis* spp.; Stefanski [82] and Giannetto and Trotti [35] for *Spirura rytipleurites seurati*; Ortlepp [65] for *Physaloptera* spp.; Quentin and Seguignes [70] for *Gongylonema* spp.; Kinsella [47] for *Angiostrongylus cantonensis*; and Gállego Franco [31] for *Brachylaima* spp.

### DNA isolation and PCR amplification

Total DNA was extracted using an NZY Tissue gDNA Isolation kit (NZYtech, Lisbon, Portugal), and DNA concentration was determined using a spectrophotometer (ND-2000 NanoDrop, Thermo Fisher Scientific, Waltham, MA, USA). PCR amplifications were carried out on samples with a DNA concentration of > 10 ng/μL using the Veriti system (Applied Biosystems, Foster City, CA, USA). Folmer *et al.*’s primers [28] were employed to amplify a fragment of the *cytochrome c oxidase subunit* 1 (COI) gene region, which is commonly used for DNA barcoding (molecular identification). A second set of primers was used for specimens that could not be amplified with the first set. Different sets of primers were tested to amplify the parasites, if one set failed, the next set was attempted in the following order: LCO1490/HCO2198, JB4.5/JB3, LCO1490/JB4.5, JB3/ CO1-R trema. The primers used for COI PCR amplification are listed in Table 1.

**Table 1:** Parasites, GenBank accession numbers of sequences and primer sets used for PCR amplification of the COI gene region.

Species	Accession number	Primers	Primers reference
*Moniliformis saudi*	OQ078755	LCO1490 (5′–GGTCAACAAATCATAAAGATATTGG–3’)	[28]
*Crenosoma striatum*	OQ078756a	HCO2198 (5’–TAAACTTCAGGGTGACCAAAAAATCA–3’)	
*Gongylonema* sp.	OQ078757–OQ078758		
*Mathevotaenia* sp.	OQ078759	JB4.5 (5’–TAAAGAAAGAACATAATGAAAATG–3’)	[11]
*Eucoleus* sp.	OQ078760	JB3 (5’–TTTTTTGGGCATCCTGAGGTTTAT–3’)	
*Aonchotheca erinacei*	OQ078761		
*Physaloptera immerpani*	OQ078762		
*Spirura rytipleurites seurati*	OQ078763–OQ078764		
*Plagiorhynchus cylindraceus*	OQ078765	LCO1490 (5’–GGTCAACAAATCATAAAGATATTGG–3’)	[11, 28]
		JB4.5 (5’–TAAAGAAAGAACATAATGAAAATG–3’)	
*Brachylaima* sp.	OQ078766	JB3 (5’–TTTTTTGGGCATCCTGAGGTTTAT–3’)	[11, 58]
		CO1-R trema (5’–CAACAAATCATGATGCAAAAGG–3’)	

We were unable to use the same nematode specimens for both morphological and molecular analyses because the latter cannot be conducted on specimens previously treated with lactophenol. This limitation represents a significant constraint for our study. The use of lactophenol, while essential for preserving and observing morphological features, renders the DNA unsuitable for subsequent molecular analysis.

The PCR reaction volume was 50 μL, consisting of 2 μL template DNA and 48 μL of PCR mix: 2 μL of each primer 10 μM, 25 μL of Taq master mix (Supreme NZYTaq II 2× Green Master Mix), 2 μL MgCl_2_ 50 mM, and 17 μL of MilliQ water. PCR conditions were as follows: 95 °C for 3 min; 35 cycles of 95 °C for 30 s, 50 °C for 30 s and 72 °C for 1 min; and finally, 72 °C for 10 min. PCR products were visualised on a 2% agarose gel and subsequently purified using an NZYGelpure kit (NZYtech). The purified products were then sent to an external company (Sistemas Genómicos S.L., Valencia, Spain) for bi-directional sequencing.

### Sequence analyses and molecular characterisation

Sequences were analysed and aligned using CodonCode v.9.0.1 (CodonCode Corporation, Dedham, MA, USA) and compared against the GenBank database (https://blast.ncbi.nlm.nih.gov/). DNA sequences obtained were deposited in GenBank under accession numbers OQ078755–OQ078766.

To identify helminths with challenging morphology, a pairwise distance matrix was constructed using the Kimura 2-parameter substitution model (K2P) in MEGA 6 software [84]. Representative COI sequences from species within each genus were obtained from GenBank. For each haplotype, one sequence was included in the K2P analysis. All available sequences of Capillariidae species for the same target region were retrieved for phylogenetic reconstruction. A maximum likelihood phylogenetic tree was constructed using MEGA 6 (bootstrap test: 500 replicates).

### Seasonality in parasite prevalence

We used Generalised Linear Models (GLMs; link function: logit; error distribution: binomial) to evaluate the impact of seasonality on the prevalence of each parasite species, based on the dates hedgehogs were presented to the COFIB hospital. We compared models that included and excluded seasonal effects in autumn (October–December), winter (January–March) and spring (April–June), on parasite prevalence among the hedgehogs that were necropsied at the COFIB wildlife hospital. We assessed the alternative models for each parasite prevalence using likelihood ratio tests (LRT). A *p*-value < 0.05 was considered statistically significant. These analyses were conducted in RStudio 4.1.2 [73].

## Results

Records from the COFIB wildlife hospital for the period 2019–2022 indicate that *A. algirus* comprised 7–9% of the hospitalised animals. Among these hedgehogs, 42–46% were successfully reintroduced to their natural habitat after rehabilitation, while 20–25% died during hospitalisation and 15–20% were euthanised for humane reasons. Through a comprehensive morphological examination and complementary molecular characterisation, we identified eleven helminth taxa infecting 135 necropsied hedgehogs (File S1). Results are summarised in Table 2.

**Table 2:** Summary of the results obtained in this study and literature review of studies published on helminth parasites associated with Atelerix algirus. Some studies were focussed on specific groups of parasites only.

Reference, hedgehog location and number of animals analysed per study.															
	This study	García-Salguero *et al.* 2019 [33]	Delgado-Serra *et al.* 2016 [21]	Paredes-Esquivel *et al.* 2019 [67]	Esteban *et al.* 1987 [27]	Esteban *et al.* 1987 [27]	Mas-Coma *et al.* 1993 [57]	Mas-Coma *et al.* 2000 [55]	Dollfus 1954 [25]	Khaldi *et al.* 2012 [46]	Torres-Martínez 1988 [87]	Mas-Coma 1979 [54]	Mas-Coma & Feliu 1977 [56]	Sánchez Vicente 2013 [76]	Fuentes *et al.* 2000 [29]
	Mallorca islands	Mallorca islands	Mallorca islands	Mallorca islands	Formentera islands	Mallorca, Menorca & Ibiza islands	Cabrera island	Ibiza island	Western Morocco	Algeria	Catalunya (Spain)	Catalunya (Spain)	Catalunya (Spain)	Canary Islands	Valencian Region (Spain)
Helminth species		*n* = 277	*n* = 7	*n* = 2	*n* = 44	*n* = 20	*n* = 1	*n* = 8	*n* = ?	*n* = 25	*n* = 2	*n* = 3	*n* = ?	*n* = 7	*n* = 3
Acanthocephala															
* Acanthocephala* sp. *larvae*										20.0%					
* Moniliformis*			14.28%		43.18%	x	x	x		32.0%					
* Moniliformis saudi*	21.84%														
* Plagiorhynchus cylindraceus*	13.79%	2.53%													
Cestoda															
* Cestoda* sp.			28.57%							4.0%					
* Mathevotaenia* sp.	10.34%														
* Mathevotaenia aethechini*									x						
* Mathevotaenia erinacei*										8.0%				42.8%	
Nematoda															
* Angiostrongylus cantonensis*	11.46%			x											
* Aonchotheca* sp.			42.85%												
* Aonchotheca erinacei*	27.59%				36.36%	x				4.0%					
* Capillariidae*			28.57%												
* Capillaria annulosa*													x		
* Crenosoma striatum*	88.07%		85.71%			x				4.0%				14.3%	66.67%
* Eucoleus* sp.	7.34%														
* Eucoleus aerophilus*			42.85%												
* Gongylonema* sp.	20.31%														
* Gongylonema* sp.					13.63%	x				36.0%					33.33%
* Physaloptera dispar = P. clausa*					93.18%	x	x	x		64.0%					
* Physaloptera immerpani*	5.48%														
* Physaloptera* sp. *larvae*										36.0%					
* Pterygodermatites plagiostoma*								x		4.0%				14.3%	33.33%
* Spirura rytipleurites seurati*	5.4%									24.0%				42.8%	
* Trichuridae gen.* sp.											50.0%				
Trematoda															
* Brachylaima* sp.	3.45%					x								28.6%	
* Dollfusinus frontalis*					11.36%			x							
* Zonorchis* sp.					2.27%										
* Zonorchis guevarai*												x			

x presence of the parasite but prevalence not quantified.

### Nematodes

**Figure 1:**
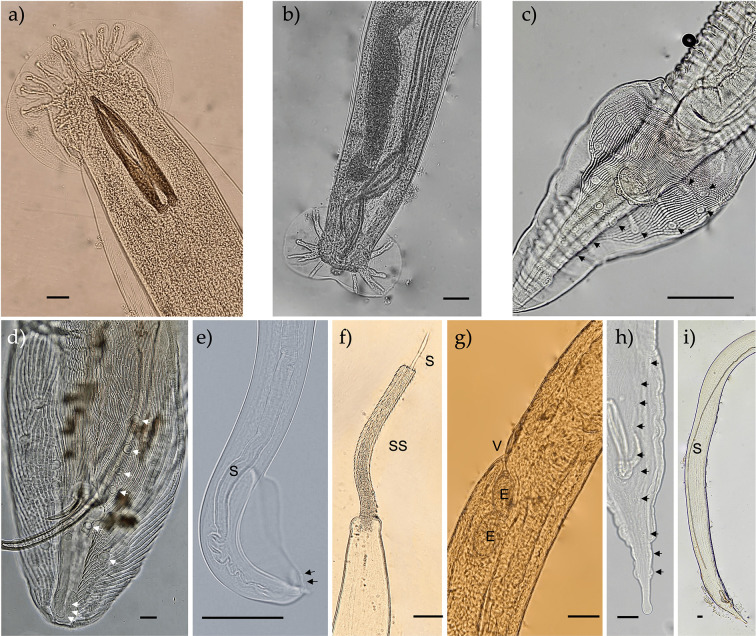
Nematodes from *Atelerix algirus* from Mallorca. Photos a and b) Typical morphology of the copulatory bursa, rays, and spicules of *Angiostrongylus cantonensis* and *Crenosoma striatum*, respectively. c) Posterior end of a *Physaloptera immerpani* male showing wide caudal alae and papillae. d) Posterior end of a *Spirura rytipleurites seurati* male specimen showing spicules, caudal alae and typical disposition of caudal papillae. e) Posterior end of an *Aonchotheca erinacei* male showing the alae and the two pairs of caudal ventrally pointed papillae. f and g) *Eucoleus* sp. male posterior end showing spicules, and female vulvar region, respectively. h and i) Posterior end of a *Gongylonema* sp. male showing the typical disposition of papillae, and long spicule, respectively. Scale bar: 50 μm. Arrows point to the papillae. S, spicule; SS, spicular sheath; V, vulva; E, egg.

The morphological and molecular analyses confirmed the identity of two metastrongylid species previously reported from North African hedgehogs in Mallorca: *Angiostrongylus cantonensis* and *Crenosoma striatum* found in the brain and lungs, respectively (Figs. 1a, 1b). These parasites were easily identified based on the available morphological descriptions [26, 47, 83, 88], while their sequence identity was confirmed by a 100% similarity to sequences already published in GenBank. Among the 11 hedgehogs infected with *A. cantonensis*, ten exhibited the typical neurological manifestations characterised by Delgado-Serra *et al.* [22]. One individual was asymptomatic for neurological signs. The lungworm *C. striatum* was the most prevalent among all helminth taxa (96/109 hedgehogs).

Using morphological keys, three other species of nematodes were identified to the species level: *Physaloptera immerpani*, *Spirura rytipleurites seurati* and *Aonchotheca erinacei* (Figs. 1c–1e). The sequences submitted to GenBank constitute the first *COI* records for these species and are the only available DNA data for the first two.

The stomach-dwelling species *P. immerpani*, found in 4/73 of the hedgehogs, displayed morphological similarities to *Physaloptera dispar*, the typical *Physaloptera* species reported in hedgehogs [46]. Our specimens showed “lips with a pair of cephalic papillae and massive external tooth”. Males showed “wide caudal alae covered by crests aligned longitudinally” as described by Ortlepp [65]. The distinguishing feature between *P. immerpani* and *P. dispar* is the third pair of post-cloacal papillae, which is sessile in the former (Fig. 1c) and pedunculate in the latter. *Spirura rytipleurites seurati* was collected from the hedgehogs’ oesophagi (3/74, 4.05%) and stomachs (1/73, 1.37%). This nematode was identified based on the typical disposition and number of the pre (4 pairs) and post (2 pairs) anal papillae (Fig. 1d) as described by Stefanski [82] and Giannetto *et al.* [35]. Finally, *Aonchotheca erinacei* (Fig. 1e) was found in 24/87 (27.6%) hedgehog intestines. This species was identified by the typical caudal wings and ventrally curved papillae, as well as the long spicule with a sheath showing transverse rings in males. The cuticular expansion of the female vulvar region also fit previous species descriptions by Hoa [38].

Lungworms of the genus *Eucoleus* (prevalence 8/109 lungs, 7.34%) were identified only to genus level because of the limited number of male specimens and their state of preservation. Nevertheless, the available male specimens show a rudimentary pseudobursa, lacking caudal lateral alae, with a spicular sheath that is long and densely covered by spines (Fig. 1f). Female specimens displayed a non-protuberant vulva (Fig. 1g). These characters correspond to those described for the genus *Eucoleus* [80]. Furthermore, the closely related *Eucoleus* and *Capillaria* genera were also differentiated based on their specific location within the hosts, with the former being found in the respiratory tract, mouth and stomachs and latter only found in the intestines [61, 80].

*Eucoleus aerophilus* and *Eucoleus boehmi* sequences used for molecular identification were obtained from [16]. These showed only 87–88% similarity to our specimens. The resulting phylogenetic tree confirmed these results, with our specimens not placed in a monophyletic clade with other *Eucoleus* species. However, the bootstrap values in some of the nodes were not statistically significant, permitting no further conclusions (File S2).

Similarly, *Gongylonema* specimens collected from 13/74 (17.57%) of the hedgehogs’ oesophagi and 2/73 (2.74%) of stomachs could only be identified at the genus level. Morphologically, our specimens appeared to belong to *G. pulchrum* as described by Quentin and Seguignes in 1979 [70]. They show the typical tail with two caudal wings, two very unequal spicules, with a very long left spicule and a short and robust right spicule and 6 and 4 pairs of pre-anal and post-anal papillae, respectively (Figs. 1h, 1i). However, the sequence-based identification analysis showed that they are genetically distant (K2P distance > 16) from *G. pulchrum*, as well as from *G. aegypti*, *G. nepalensis* and *G. neoplasticum* (Table 3); therefore, the species identity of the specimens remains unresolved.

**Table 3:** Pairwise genetic distances (K2P) between Gongylonema species (COI sequences) available in GenBank and those obtained in this study. Sequences retrieved from GenBank are indicated with the accession numbers.

Species	1	2	3	4	5	6
1. *Gongylonema* sp. Haplotype 1 OQ078757 (this study)	–					
2. *Gongylonema* sp. haplotype 2 OQ078758 (this study)	0.91	–				
3. *G. aegypti* LC026046	21.77	21.45	–			
4. *G. neoplasticum* LC334451	21.86	21.86	9.51	–		
5. *G. pulchrum* AP017685	16.27	16.86	14.22	13.10	–	
6. *G. nepalensis* LC278393	18.97	19.28	14.51	13.69	10.30	–
7. *Gongylonema* sp. LC612845	17.42	18.02	16.72	15.29	12.91	12.90

### Platyhelminthes (Trematoda and Cestoda)

We identified two platyhelminth parasites, the trematode *Brachylaima* sp. and the cestode *Mathevotaenia* sp., found in 3/87 and 9/87 of the small intestines, respectively. Specimens were identified to the genus level using the available morphological keys [31]. When comparing the resulting COI sequences, no similar DNA records were found in the GenBank database.

**Figure 2:**
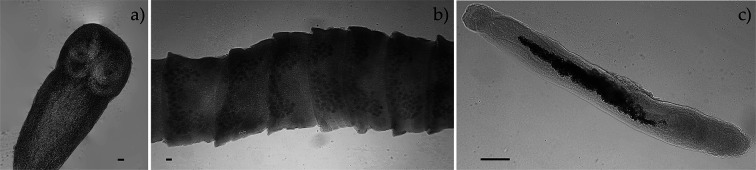
Platyhelminths found in *Atelerix algirus* from Mallorca. a) *Mathevotaenia* sp. detail of the unarmed scolex with four oval suckers. b) Stained proglottids of *Mathevotaenia* sp. showing the distribution of the testes. c) Stained *Brachylaima* sp. Scale bar: 50 μm.

The cestode’s morphological features were consistent with those of *Mathevotaenia* [77]: an unarmed scolex bearing 4 oval suckers, craspedote proglottids with genital pores alternating irregularly and situated in the anterior half of the proglottid, 40–50 testes in the posterior part of the proglottid, body width 1 mm and length up to 130 mm (Figs. 2a, 2b). The cestode found in this study does not correspond to *M. erinacei* as the total length does not match the original description.

The morphology of the trematode was consistent with that of *Brachylaima* sp. [31]: body flattened and oval, with testes arranged in tandem at the posterior end of the body, and intertesticular ovary, somewhat sinuous ceca and an acetabulum close to the mid-body (Fig. 2c).

### Thorny-headed worms (Acanthocephala)

**Figure 3:**
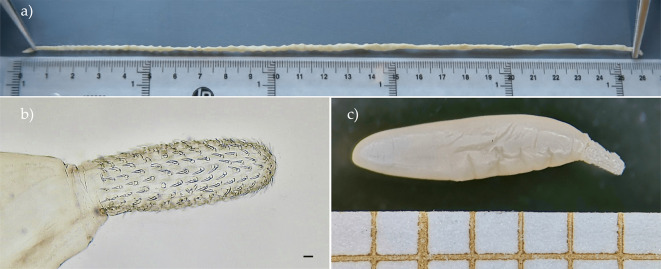
Acanthocephalans found in *Atelerix algirus* from Mallorca. a) Abnormally large *Moniliformis* s*audi.* b and c) Proboscis and whole body of an immature *Plagiorhynchus cylindraceus*. Scale bar: 50 μm.

Two acanthocephalan parasites were found infecting the small intestines of the hedgehogs: *Moniliformis saudi* and *Plagiorhynchus cylindraceus*, with the former being more prevalent than the latter (21.84% *vs.* 13.79%, respectively). Some adult *M. saudi* specimens were more than twice the size (261 mm) of those previously described by Amin *et al.* [4] (117.5 mm maximum length) (Fig. 3a). A single haplotype was found in the two specimens that were sequenced. The COI sequence analysis showed a 99.69% similarity with previously described *M. saudi* [4, 18] and 99.01% with *Moniliformis cryptosaudi* [5]. Genetic distances calculated with the Kimura-2-parameter (K2P) model showed that intrageneric distances varied from 0.83% with *M. saudi* to 33.4% with *Moniliformis* (Table 4)*,* which is the only *Moniliformis* species previously reported in European hedgehogs. *Plagiorhynchus cylindraceus* specimens were found as cystacanths (Figs. 3b, 3c) and the single haplotype found was 99.83% similar to those previously reported in Mallorca [33].

**Table 4:** Pairwise genetic distances (K2P) between all COI sequences from Moniliformis species available in GenBank and those obtained in this study. Sequences retrieved from GenBank are indicated with the accession number.

Species	1	2	3	4	5	6	7	8	9
1. *M. saudi* (OQ078755, this study)	–								
2. *M. saudi* KU206783	0.83	–							
3. *M. cryptosaudi* MH401041	1.25	0.41	–						
4. *M. moniliformis* MT512678	33.40	32.72	33.40	–					
5. *M. moniliformis* AF416998	33.40	32.72	33.40	0.00	–				
6. *Moniliformis* sp. OK415026	18.81	18.81	18.81	28.73	28.73	–			
7. *M. necromys*i MT803593	31.43	31.43	32.10	36.07	36.07	32.10	–		
8. *M. ibunami* MW115575	30.94	31.61	30.94	35.57	35.57	28.98	33.29	–	
9. *M. ibunami* MW115576	30.94	31.61	30.94	35.57	35.57	28.98	33.29	0.00	–
10. *M. kalahariensis* MH401040	28.44	29.09	29.74	37.44	37.44	32.84	30.82	26.83	26.83

The lungs were the most affected organs, with 88.07% infected by lungworms. No parasites were found in the nasal sinuses, heart, kidney, liver or gallbladder.

Coinfections in the lungs with *C*. *striatum* and *Eucoleus* sp. were present in 8 of the 109 (7.34%) samples examined. Only one oesophagus was infected with two parasite species: *Gongylonema* sp. and *Spirura rytipleurites seurati*. Ten of 87 (11.49%) intestines were coinfected, as follows: one by *A. erinacei* and *Mathevotaenia* sp., four by *M. saudi* and *A. erinacei*; one by *M. saudi* and *P. cylindraceus*; one by *M. saudi*, *P. cylindraceus* and *A. erinacei*; one by *A. erinacei* and *P. cylindraceus*; one by *A. erinacei* and *Brachylaima* sp. and one by *Mathevotaenia, Brachylaima* and *A. erinacei.*

### The effect of seasonality on helminth prevalence

**Figure 4:**
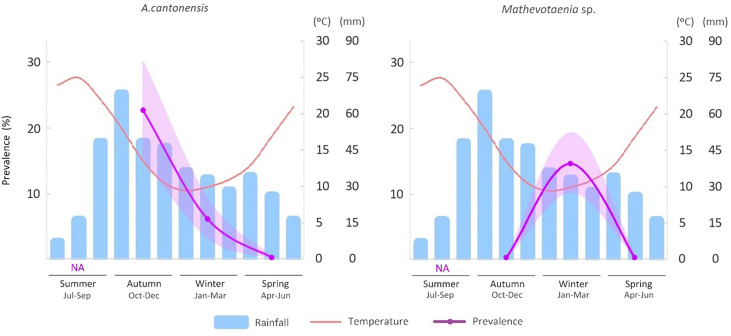
Seasonal prevalence (and standard error, represented as SE) of *A*. *cantonensis* and *Mathevotaenia* sp. in hedgehogs subjected to necropsies (purple lines). The figure also shows the mean monthly temperature (red line) and rainfall (blue bars) in Mallorca during the period 1971–2000 (data from *Agencia Estatal de Meteorología* AEMET).

Table 5 summarises the results of the GLM analysis to identify for each parasite whether the constant or seasonal model was better. *Angiostrongylus cantonensis* and *Mathevotaenia* sp. exhibit a significant LRT *p*-value for seasonal prevalence, indicating that their prevalences fluctuate throughout the year depending on the season, being highest in autumn and winter, respectively (Fig. 4). For all other parasites, the constant model is better, indicating that no significant differences in prevalence were detected between the seasons.

**Table 5:** GLM models testing the extent of seasonality in the prevalence of the different parasites studied. Notation: df – degrees of freedom, AIC – Akaike Information Criteria, Dev – Deviance, LTR *p*-value – Likelihood Ratio Tests *p*-value. The best model explaining the prevalence of each parasite is in bold.

Parasite	Model	df	AIC	Dev	LRT p-value
*Angiostrongylus cantonensis*	Constant	1	70.350	68.350	
	**Seasonal**	**3**	**67.850**	**61.850**	0.038
*Aonchotheca erinacei*	**Constant**	**1**	**104.487**	**102.490**	
	Seasonal	3	107.892	101.890	0.743
*Brachylaima* sp.	**Constant**	**1**	**28.099**	**26.099**	
	Seasonal	3	31.626	25.626	0.789
*Crenosoma striatum*	**Constant**	**1**	**81.670**	**79.670**	
	Seasonal	3	82.267	76.267	0.182
*Eucoleus* sp.	**Constant**	**1**	**59.188**	**57.188**	
	Seasonal	3	61.425	55.425	0.414
*Gongylonema* sp.	**Constant**	**1**	**82.363**	**80.363**	
	Seasonal	3	84.313	78.313	0.359
*Mathevotaenia* sp.	Constant	1	59.871	57.871	
	**Seasonal**	**3**	**56.063**	**50.063**	**0.020** ^*^
*Moniliformis saudi* ^+^	**Constant**	**1**	**93.326**	**91.326**	
	Seasonal	3	96.384	90.384	0.624
*Physaloptera immerpani* ^+^	**Constant**	**1**	**34.450**	**32.450**	
	Seasonal	3	36.990	30.990	0.481
*Plagiorhynchus cylindraceus*	**Constant**	**1**	**72.681**	**70.681**	
	Seasonal	3	74.019	68.019	0.264
*Spirura rytipleurites seurati*	**Constant**	**1**	**34.450**	**32.450**	
	Seasonal	3	36.990	30.990	0.481

* Statistically significant effects of seasonality (LRT *p*-values < 0.05) are indicated with an asterisk.

+ First report in *Atelerix algirus*.

## Discussion

Pathogen surveillance plays a crucial role not only in identifying potential threats to wildlife, but also in assessing the circulation of zoonotic agents that could potentially spill over to human populations. This study represents the most comprehensive survey of helminth parasites in the hedgehog species *Atelerix algirus*, which is widely distributed in the Mediterranean region. Our approach involved both morphological and molecular (COI) characterisation of 11 helminth taxa, in addition to shedding light on the seasonality of rat lungworm (*A. cantonensis*) prevalence in the region.

*Angiostrongylus cantonensis* serves as the main aetiological agent of eosinophilic meningitis in humans and other vertebrates, often leading to severe clinical manifestations, particularly motor impairments [51, 81]. In 2018, this zoonotic nematode was reported for the first time in Mallorca in hedgehogs [67] and has since continued to spread across the island [22]. In 2022, *A. cantonensis* was also detected in urban rats in continental Spain [30]. Despite its potential threat to wildlife and humans, there is a lack of systematic surveillance of this parasite. This study represents the first effort to understand the prevalence of this pathogen in a wildlife species. We recorded an overall prevalence of 11.46% in *A. algirus* hedgehogs from Mallorca. As previously stated, this prevalence is based on the 96 brains examined, as some were excluded due to damage.

It is challenging to establish *A. cantonensis* prevalence comparisons with previous studies on accidental hosts since they have focussed solely on fauna exhibiting signs of neurological disease. The only comparable study we are aware of was conducted on tawny frogmouths (*Podargus strigoides*) in Australia [53], which found that 80% of the birds with neurological disease were infected with *A. cantonensis*. Further investigations are urgently needed to understand the threat this nematode may pose to European wildlife. However, an equally significant discovery of our study was the identification of an asymptomatic case of angiostrongyliasis in an infected hedgehog. To the best of our knowledge, this constitutes the sole confirmed report of a subclinical *A. cantonensis* infection in non-human accidental hosts. In humans, asymptomatic neuroangiostrongyliasis cases have been reported previously [74].

The results of the GLM analysis reveal a distinct seasonal pattern for neuroangiostrongyliasis on Mallorca, with most cases occurring in autumn (October to December) and to a lesser extent during winter (January to March) (Fig. 4). These findings are consistent with a prior study conducted on dogs in Australia, where *A. cantonensis* also circulated primarily during the same seasons, *i.e.* periods of increased humidity and average daily temperatures of 17–25 °C [51]. Our results are also consistent with observations made in Europe regarding the seasonality of the congeneric species *Angiostrongylus vasorum* [63]. During autumn, Mallorca experiences mild temperatures and maximal rainfall levels, conditions that enhance the abundance and availability of gastropods, the intermediate hosts of *Angiostrongylus* parasites. This in turn increases the likelihood of hedgehogs consuming infected gastropods, potentially elevating their *A. cantonensis* infection prevalences (Fig. 4).

The development of *A. cantonensis* larvae within gastropods ceases when temperatures fall below 15 °C [52]. Also, when experimentally infected, the viability of *A. cantonensis* L3 larvae in slugs was reduced when exposed to winter temperatures [7]. These studies are consistent with the reduced prevalence observed during winter months (Fig. 4). The seasonal pattern of the disease may suggest that infected hedgehogs do not survive the winter. To comprehensively evaluate the detrimental impact of this pathogen on hedgehogs, long-term studies are imperative, especially considering reports of declining populations [32]. Additionally, future investigations should encompass data from the summer months and incorporate larger sample sizes to gain a deeper understanding of the seasonal incidence of this parasite.

This study also revealed a high prevalence (88.07%) of the lungworm *Crenosoma striatum* in *Atelerix algirus*. This gastropod-borne nematode has previously been reported in *A. algirus* populations across various regions, including the Balearic Islands, mainland Spain, the Canary Islands and Algeria (Table 2). It is also highly prevalent in the European hedgehog, *E. europaeus* [90]. However, the epidemiology of this disease in *A. algirus* had not been thoroughly investigated until now. Previous studies on this subject have typically involved only a few specimens (<10), apart from a study in Algeria (Table 2), which recorded a much lower prevalence (4%) despite Algeria being the native range of this lungworm [46]. *Crenosoma striatum* inhabits the trachea, bronchi and bronchioles, leading to airway occlusion and often causing cardiorespiratory problems and granulomatous pneumonia [9], as well as increasing the risk of anaphylaxis and hyperpnoea [10]. In other regions, there are reports of severe and fatal cases during autumn in *E. europaeus* [19]. While we did not find evidence of seasonality in this infection, further studies are necessary to understand the impact of climate on the disease.

Molecular characterisation plays a pivotal role in enhancing helminth species identification and providing comprehensive insights into the life cycles of these parasites (*e.g.* intermediate hosts) [33]. In this study, we have conducted molecular characterisations of three nematode species for the first time: *Spirura rytipleurites seurati*, *Aonchotheca erinacei* and *Physaloptera immerpani.* We anticipate that our study will enhance surveillance efforts and clarify species identities in future studies. Particularly noteworthy is *Physaloptera immerpani*, which has remained relatively obscure since its initial description in 1937 when it was found in the stomach of *Atelerix frontalis* in South Africa [65]. Given its morphological resemblance to *Physaloptera dispar*, we postulate that some of the stomach parasites previously reported in hedgehogs may have been erroneously misidentified as *P. dispar* (Table 2). *Physaloptera* species have previously been reported sporadically in humans; however, precise species-level identification has not always been feasible [79].

We identified some specimens morphologically as *G. pulchrum* [70]. However, the K2P pairwise analysis of the COI gene region revealed a substantial genetic distance (16.27%–16.86%) between our specimens and the recognised *G. pulchrum* (Table 3). This is higher than the intraspecific divergence reported for nematodes (up to 5%) [24]. Therefore, the identity of our *Gongylonema* specimens remains unresolved. The COI gene region has been often used to detect hidden diversity in other taxa [23]. In the case of parasites, the detection of cryptic species holds particular significance, as these variants may differ in terms of pathogenicity, host specificity and resistance to anthelmintic treatments [17]. Given the numerous documented cases of gongylonemiasis in humans [50], elucidating the species composition within the *Gongylonema* genus is a crucial step prior to identification of potentially zoonotic agents.

Two thorny-headed worms were found infecting *A. algirus* in Mallorca: *Moniliformis saudi* and *Paraechinus cylindraceus*. Notably, our identification of *M. saudi* marks the first report of this parasite in *A. algirus*. Despite the considerable size difference compared to those described in other regions, the morphology of our specimens closely matched the established description of this helminth [4]. Furthermore, the genetic distance between our specimens and *M. saudi* was 0.83% (K2P model), a value well within the range of intraspecific variation reported for acanthocephalans (1–5%) [34]. *Moniliformis saudi* had previously only been documented in *Paraechinus aethiopicus* in Saudi Arabia, while previous studies in Europe had reported *Moniliformis moniliformis* in *E. europaeus* [43] and *Atelerix algirus* from the Balearic Islands [21, 27, 55, 57]. These reports were based on morphological identifications, which can occasionally lead to errors in helminth identification [64]. Given the substantial morphological similarity between *Moniliformis* species, we postulate that many of these prior reports might, in fact, correspond to *M. saudi*. The accurate identification of this parasite species has important implications for public health as *M. moniliformis* is a known zoonotic agent [75], while such zoonotic status has not been demonstrated for *M. saudi.* Given the resemblance in the life cycles of these species, it is reasonable to suspect that *M. saudi* could also be zoonotic, although further investigations are necessary to confirm this.

*Plagiorhynchus cylindraceus* was detected in the peritoneum and intestines of the hedgehogs. This species is a bird parasite and has been reported in other hedgehog species: *Erinaceus europaeus* from Germany, the United Kingdom, New Zealand [78], the Czech Republic [69] and *Erinaceus roumanicus* from the Czech Republic [69]. Skuballa *et al.* [78] suggested that insectivores and other small mammals, such as hedgehogs, could serve as paratenic hosts, facilitating the transmission of this parasite to birds of prey. The pathogenicity of acanthocephalans can vary depending on factors such as parasite burden and the extent of host tissue penetration, often leading to secondary infections [78]. The observed ability of *P. cylindraceus* to penetrate the intestinal wall coupled with the increased prevalence noted in this study compared to previous surveys in the same area [33] suggests that this species may warrant further consideration as a potential emerging concern for *A. algirus* in Mallorca.

The only cestode reported infecting *A. algirus* in Spain (Canary Islands) is *Mathevotaenia erinacei* [76]. However, *M. aethechini* has also been reported infecting *A. algirus* in Morocco [77]. In this study, we could only classify our specimens at the genus level, as *Mathevotaenia* sp. This parasite exhibited a significantly high prevalence during the winter (Fig. 4). The reduced availability of food resources during winter, combined with the fact that in Mallorca hedgehogs enter a state of partial hibernation during this period, may predispose them to infection by this cestode. However, cestodes generally cause mild infections [20], which does not align with our findings that suggest a more acute and potentially lethal course of the disease. In humans, *Mathevotaenia* infections have been reported, but appear to be exceedingly rare [49]. Identification of the trematode *Brachylaima* sp. to the genus level was validated through both morphological and molecular techniques. Butcher and Grove [12] documented human infections caused by *Brachylaima cribbi* in Australia, a parasite known to induce chronic infections that can persist for up to 18 months; it is typically transmitted through the ingestion of raw or undercooked gastropods [13].

We anticipate that the molecular characterisation of helminth parasites of *A. algirus* in Mallorca will significantly contribute to their early detection in other regions of the world. Furthermore, it promises a more comprehensive understanding of the life cycles and distributions of these pathogens. Equally important is the understanding of the seasonal dynamics of these parasites, particularly in the case of the zoonotic nematode *Angiostrongylus cantonensis*. The autumn/winter seasonal pattern observed for the rat lungworm in Mallorca, provides valuable and novel information for public health authorities in the region. Understanding the dynamics of these pathogens is essential for developing effective prevention and control strategies. We expect this study will facilitate future surveillance efforts, ultimately leading to improved management and control of helminth infections in hedgehog populations, including those with zoonotic potential.

## Supplementary materials

The supplementary material of this article is available at https://www.parasite-journal.org/10.1051/parasite/2024069/olm. *File S1*. Helminth prevalence in hedgehogs arriving at the wildlife recovery centre in Mallorca. *File S2*. Maximum likelihood phylogenetic tree based on the *cytochrome oxidase* I gene region. This shows the location *Eucoleus* sp. found in this study in relation to Capillariidae nematodes sequences available in GenBank.
