# Supplementary material for: Helminth diversity and seasonality of Angiostrongylus cantonensis in hedgehogs from Mallorca
Source: Parasite. 2024 Nov 6;31:69. doi: 10.1051/parasite/2024069 (PMC11540299; doi:10.1051/parasite/2024069)
Supplement: Supplementary file 1 — File S1. Helminth prevalence in hedgehogs arriving at the wildlife recovery centre in Mallorca. [file parasite-31-69-s1.pdf]

Supplement File 1

Helminth prevalence in hedgehogs examined at COFIB in Mallorca.

Helminth species	Prevalence					
	Brain (96)	Lungs (109)	Oesophagus (74)	Stomach (73)	Intestine (87)	Peritoneum (87)
**Acanthocephala**						
Moniliformis saudi					21.84%	
Plagiorhynchus cylindraceus					5.74%	8.04%
**Cestoda**						
Mathevotaenia sp.					10.34%	
**Nematoda**						
Angiostrongylus cantonensis	11.46%					
Aonchotheca erinacei					27.59%	
Crenosoma striatum		88.07%				
Eucoleus sp.		7.34%				
Gongylonema sp.			17.57%	2.74%		
Physaloptera immerpani				5.48%		
Spirura rytipleurites seurati			4.05%	1.37%		
**Trematoda**						
Brachylaima sp.					3.45%	

Numbers in brackets are the total numbers of each organ analysed.
