# Supplementary material for: Helminth diversity and seasonality of Angiostrongylus cantonensis in hedgehogs from Mallorca
Source: Parasite. 2024 Nov 6;31:69. doi: 10.1051/parasite/2024069 (PMC11540299; doi:10.1051/parasite/2024069)

Supplement File 2

Maximum likelihood phylogenetic tree based on the *cytochrome oxidase* I gene region. This shows the location *Eucoleus* sp. found in this study in relation to Capillariidae nematodes sequences available in GenBank.

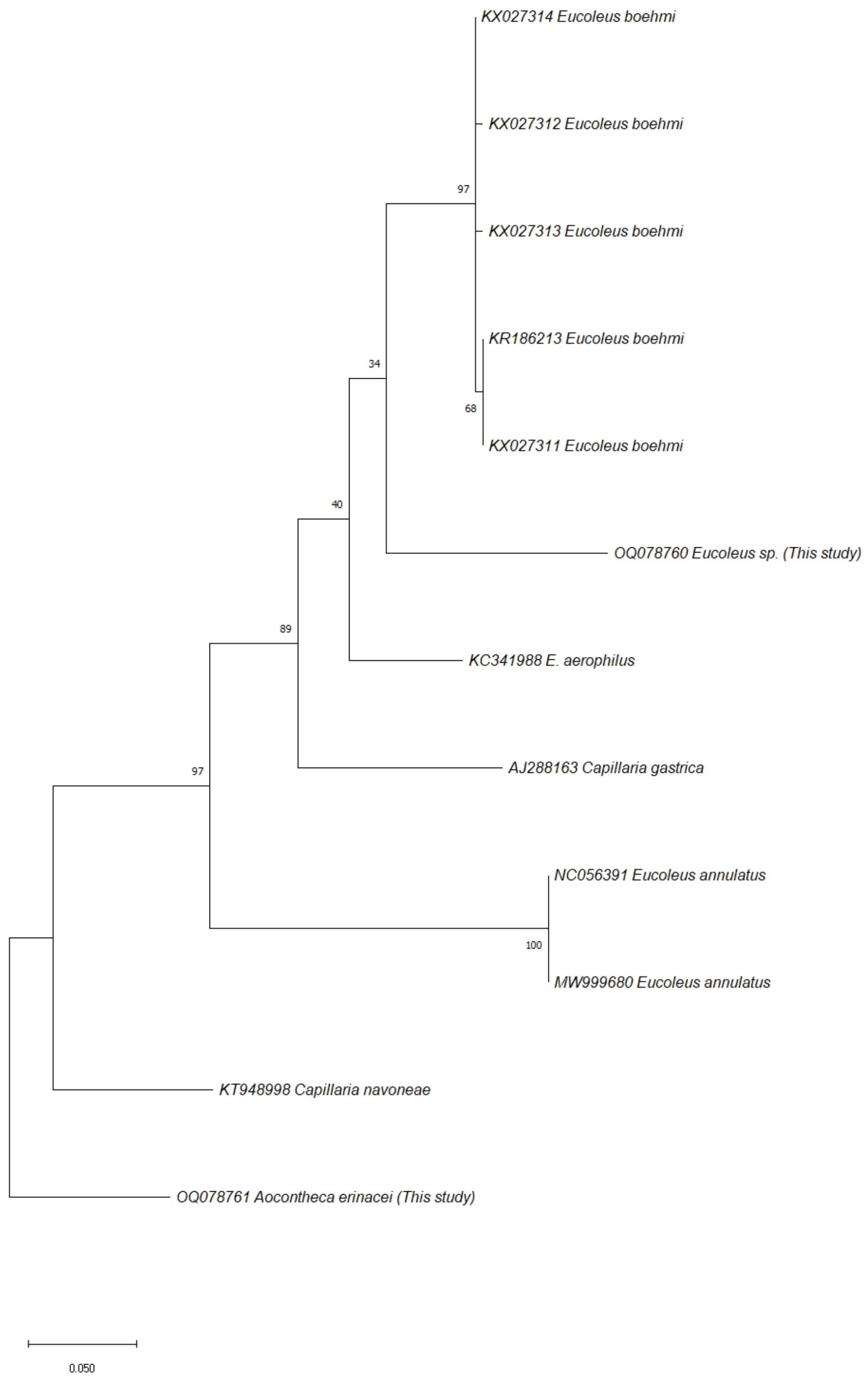

Supplement: Supplementary file 2 — File S2. Maximum likelihood phylogenetic tree based on the cytochrome oxidase I gene region. This shows the location Eucoleus sp. found in this study in relation to Capillariidae nematodes sequences available in GenBank. [file parasite-31-69-s2.pdf]
